# Different sources of the numerical comparison size effect

**DOI:** 10.3758/s13421-025-01781-2

**Published:** 2026-03-17

**Authors:** Attila Krajcsi, Petia Kojouharova

**Affiliations:** 1 Department of Cognitive Psychology, Institute of Psychology, ELTE Eötvös Loránd University, Izabella utca 46, 1064 Budapest, Hungary; 2 HUN-REN Research Centre for Natural Sciences, Institute of Cognitive Neuroscience and Psychology, Magyar tudósok körútja 2, 1117 Budapest, Hungary

**Keywords:** Approximate number system, Numerical distance effect, Numerical size effect, Discrete semantic system

## Abstract

In symbolic number comparison tasks, the numerical distance and size effects are largely influenced by the statistical properties of the stimuli. Here, we tested whether nonsymbolic number comparison is also sensitive to the statistical features of the stimuli. We found that unlike in the symbolic comparison task, in the nonsymbolic comparison task, the distance effect was mainly influenced by the values and not by the statistics of the stimuli. We also found that while the size effect was modified by the frequency of the stimuli, the change of the size effect may not have originated in the difficulty of the task (as predicted by the psychophysical models) but in other parameters of the decision process, such as the adjustment of the decision threshold. Additionally, while the symbolic size effect change is stable throughout the session, the nonsymbolic size effect change gradually decreases. These results confirm that nonsymbolic number comparison is mainly driven by a psychophysical process; however, stimulus frequency has an additional role. The results demonstrate again that symbolic and nonsymbolic numerical stimuli are compared qualitatively differently. Diffusion model parameter recovery also demonstrated that the numerical size effect is not a single effect but can have at least three different generators.

**Supplementary Information:**

The online version contains supplementary material available at 10.3758/s13421-025-01781-2.

## Introduction

### Models of elementary number processing

Elementary number processing is assumed to be a cornerstone of higher-level mathematical performance (Dehaene, 1992; Moyer & Landauer, 1967; Nieder, 2005). Simple number handling can be essential to predict or train mathematical abilities that are relevant in school and in everyday life (Halberda et al., 2008; Odic & Starr, 2018; Park & Brannon, 2013; Piazza et al., 2010).

In a number comparison task, two effects, the numerical distance and size effects are believed to be critical in identifying one of the underlying key number representations (Dehaene, 1992; Moyer & Landauer, 1967). The distance effect means better performance when the difference of the two numbers is larger, and the size effect is better performance when the numbers are smaller.

**Fig. 1:**
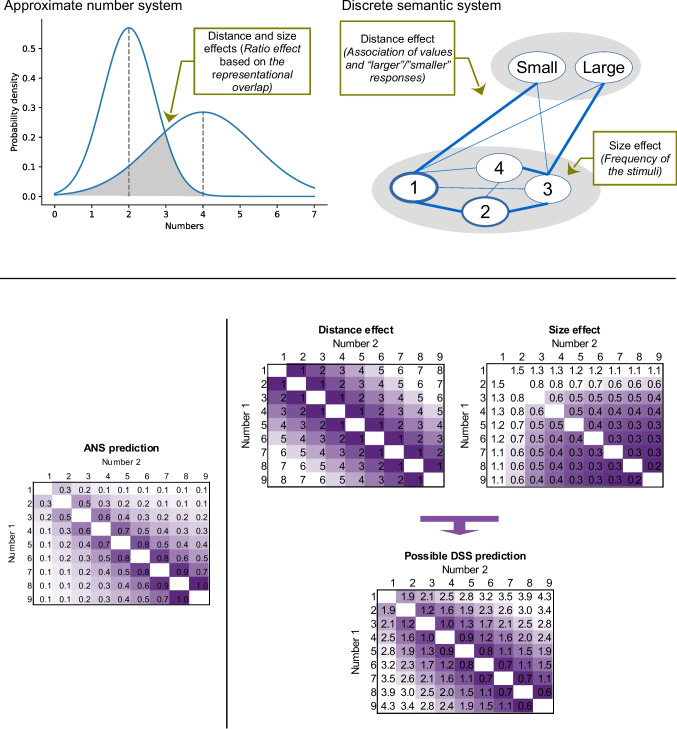
**Top row**. Possible implementations of the ANS (left) and DSS (right) models. In the ANS model, larger numbers are proportionally noisier, and comparison performance is rooted in the overlap of the two to-be-represented numbers. In the DSS model, nodes of the network are connected, the connection weights can differ (see different width of the connection lines on the figure), and the nodes can represent different frequencies (see different width of the node contour line on the figure). (The DSS account applies only to symbolic notation; see Fig. 2) **Bottom row**. Ratio effect in the ANS model (left), and the ratio-like effect in the DSS model (right) in comparison tasks. In the DSS model, distance and size effects are independent effects and their sum forms a ratio-like effect. Rows and columns are the to-be-compared numbers, cells are the predicted performance on an arbitrary scale. Darker cells mean more difficult trials (note that on arbitrary scales, more difficult trials can be expressed as either smaller or larger values). The ANS performance values are calculated as *a* × log(*large*/*distance*) + *b* in line with psychophysical models (Dehaene, 2007). The DSS performance values are calculated as *a*_1_×*distance* + *a*_2_×(*x*_1_^−1^+*x*_2_^−1^) + *b*, where *large* is the larger number, *distance* is the difference between the two numbers, *x*_1_ and *x*_2_ are the two to-be-compared numbers. The parameter *a*, *a*_1_, *a*_2_ and *b* are free parameters (the parameters *a* and *a*_2_ are set to 1, *a*_1_ to 0.4, and *b* to 0). Conceptually, the *distance* term is an assumed linear distance effect, the *x*_1_^−1^+*x*_2_^−1^ term reflects the size effect as the power function relation between the frequency of the values in everyday life and their values (Dehaene & Mehler, 1992), while the *a*_*1*_ and *a*_*2*_ parameters are set to have a reasonable proportion of the distance and size effects on the two arbitrary scales. See additional technical considerations and possible alternative formulas in Krajcsi et al. (2016). (Color figure online)

The most frequently quoted model accounting for the numerical distance and size effects assumes that the two indices reflect a single psychophysics-based ratio effect (Dehaene, 2007; Moyer & Landauer, 1967). In psychophysical models, comparison performance depends on the ratio of the two values (Fig. 1, bottom left), in line with the Weber principle.^1^ Accordingly, an evolutionarily old, noisy, analog (i.e., continuous) representation, termed the analog (or approximate) number system (ANS), is believed to generate this ratio effect, and both distance and size effects are considered to be the sign of the ANS processing (Dehaene, 2007). In a possible implementation, numbers are stored in a noisy representation where the noise is proportional to the size of the to-be-stored value, and number comparison performance (i.e., the ratio effect) is rooted in the overlap of the two to-be-compared values (Fig. 1, top left; Dehaene, 2007).

In an alternative framework, while nonsymbolic stimuli (such as arrays of dots or series of events) are processed by the ANS, in symbolic number (such as Indo-Arabic numbers or number words) processing, the distance and size effects are accounted for by a representation consisting of a network of nodes (Fig. 1. top right). This alternative representation, termed the discrete semantic system (DSS), can also produce the two comparison effects, without relying on psychophysical mechanisms (Krajcsi et al., 2016, 2022, 2024b). In the DSS model, it is assumed that the distance and size effects are independent effects, and the single ratio effect is only apparent (Fig. 1, bottom right). First, the size effect can be explained by the frequency of numbers: In everyday life, smaller numbers are more frequent than larger numbers (Dehaene & Mehler, 1992), and smaller numbers can be easier to process because they are the more frequent stimuli. This hypothesis was supported by empirical data: The presence of the symbolic size effect (but not the distance effect) was mainly influenced by the frequency manipulation of the stimuli when new artificial number symbols were used (Krajcsi et al., 2016). The size effect was modified according to similar frequency manipulation in Indo-Arabic numbers, too, in which notation, former experience had already shaped the observed frequency of those symbols (Kojouharova & Krajcsi, 2019). Second, the distance effect can be an association effect. It is possible that, based on co-occurrences, large numbers are more strongly associated with the concept or label of “large” compared with “small,” while small numbers have the opposite associations. Regarding the distance effect, numbers with a large difference may have more differing association to the concepts “large” or “small” than numbers with small difference; therefore, it could be easier to choose the larger or the smaller number when the numerical difference (and, therefore, the association strength difference) is large (see a functionally similar model in Verguts & Fias, 2004;Verguts et al., 2005). In line with this hypothesis, it was found that when association statistics were manipulated by omitting specific numbers in the session, the distance effect followed the association statistics and not the values of the numbers (Kojouharova & Krajcsi, 2018; Krajcsi & Kojouharova, 2017). According to the DSS model, combining these distance and size effect components can form a pattern that is similar to the psychophysics-based ratio effect (Fig. 1, bottom). This similarity is the reason why many former accounts assumed that the distance and size effects are the expression of a single ratio effect, however, appropriate tests reveal that the symbolic distance and size effects are independent (Hohol et al., 2020; Krajcsi, 2017; Krajcsi et al., 2022).

To summarize, for symbolic number comparison tasks, the classic ANS model assumes that the distance and size effects are rooted in the psychophysics-based ratio effect. Alternatively, the DSS model assumes that, in a symbolic number comparison task, the size and difference effects are independent, and both effects are rooted in various statistical properties of the stimuli (Fig. 1).

**Fig. 2:**
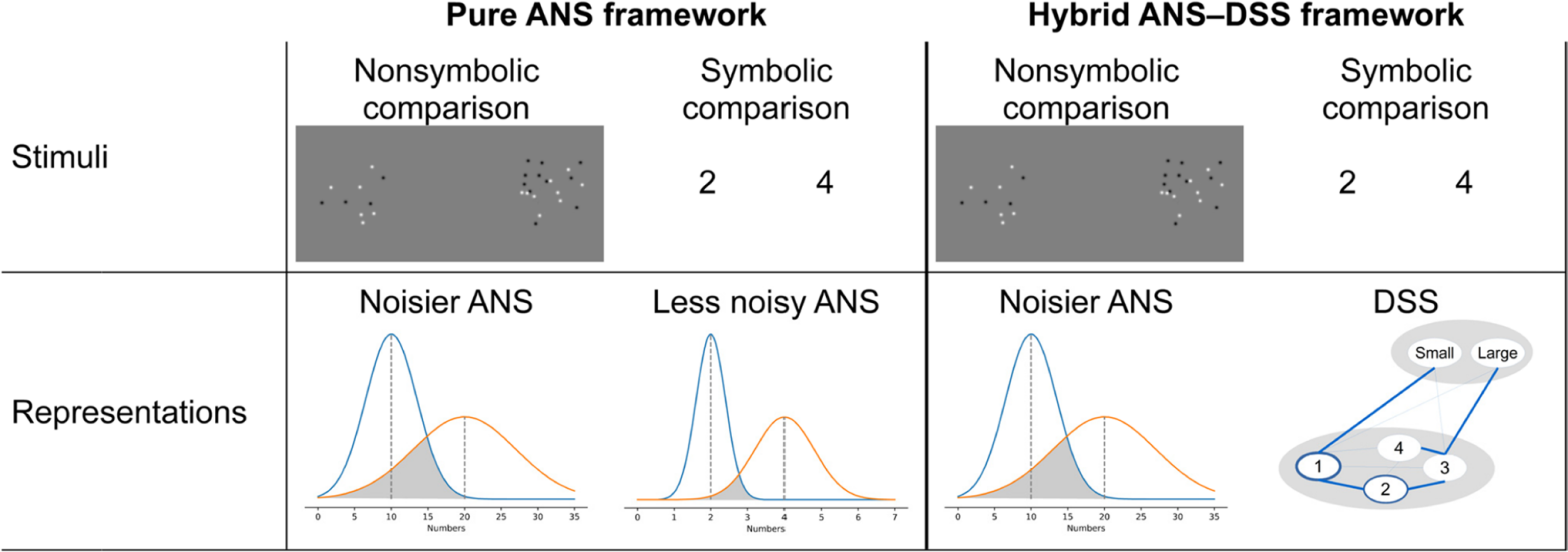
The pure ANS and the hybrid ANS–DSS frameworks’ explanation about symbolic and nonsymbolic elementary number processing

Expanding these views for nonsymbolic number comparison task, a pure ANS framework assumes that it is the ANS that supports both symbolic and nonsymbolic number processing, although the precision of the system may differ between the two types (Dehaene, 2007), which difference can explain for example, why symbolic comparison is more precise than nonsymbolic comparison. Alternatively, in the hybrid ANS–DSS framework, it is assumed that while nonsymbolic numerosities are processed by the ANS, symbolic numbers are handled by the DSS (Fig. 2). Note that, in both the pure ANS and the hybrid ANS–DSS frameworks, nonsymbolic numerosity comparison is accounted for by the ANS.

### The role of the stimulus statistics in nonsymbolic numerosity processing

Both the pure ANS and the hybrid ANS–DSS frameworks assume that nonsymbolic elementary numerical processing is backed by the psychophysical ANS. In that model, the frequency or the statistical properties of the stimuli are not relevant factors during number processing. Additionally, the empirical tests of the DSS model assume that (in line with the ANS model) the statistical properties do not influence nonsymbolic numerical processing. Still, there are various considerations why stimulus statistics in nonsymbolic numerosity processing could be influential, which considerations we discuss below. To our knowledge the effect of the stimulus statistics on nonsymbolic numerosity processing has not been tested empirically yet, even though statistical influence is possible either as an additional effect along with the psychophysical ratio effect, or even as a modulator of the psychophysical process. If statistical effects can be demonstrated in nonsymbolic number processing, this finding may lead to reconsidering the ANS model, or more generally, the psychophysical models. Discovering statistical effects also influences how the DSS model can be appropriately tested against the ANS model.

#### Association of the values and the “small”– “large” properties

A statistical property of the numerical stimuli that may influence the comparison performance is association statistics. The association statistics between values and “smaller”– “larger” responses can form a characteristic pattern. If the stimuli frequency distribution is uniform (i.e., all stimuli are shown with the same probability), then numbers form different associations with the “large” or “small” responses. For example, if numbers between 1 and 9 are presented, then 9 is larger than the other number 100% of the time, 8 is larger 87.5% of the time, 7 is larger 75% of the time. This association statistics turned out to be essential in symbolic number comparison: When the association was manipulated, the distance effect changed accordingly (Kojouharova & Krajcsi, 2018; Krajcsi & Kojouharova, 2017). The effect is also remarkable because a seemingly neutral, that is, uniform frequency distribution of the stimuli can generate a characteristic association effect, that is, the distance effect. One can imagine that similar effects can be observed in nonsymbolic comparison too. For example, even in comparison with nonsymbolic stimuli, the response (large–small, left–right) can be symbolic and categorical. Because of this symbolic component, the performance of the nonsymbolic comparison may be sensitive to the association information.

#### The role of the frequencies of the nonsymbolic numerical stimuli

Some considerations in the literature suggest that the frequency of the stimuli can also have an effect on the processing of those stimuli.

Generally, psychophysical models or, more specifically, ANS models usually do not assume that the frequency of the stimuli in the natural environment has a substantial effect (Dehaene, 2007; Kingdom & Prins, 2010). While it is well-known that symbolic numbers are not distributed uniformly in everyday life (Dehaene & Mehler, 1992), in the pure ANS framework, it is assumed that the psychophysics-based ratio effect is the only factor that determines the comparison performance without any role of the frequency (Dehaene, 1992, 2007).^2^ Still, there are considerations that may assume a frequency-based effect. In a Bayesian framework, it is proposed that perceptual systems assume stability in the environment, and in line with this assumption, perceptual processing considers not only the present stimulus but also previous stimuli. This assumption can account for a series of phenomena in psychophysics (e.g., Petzschner et al., 2015). Considering the previous stimuli is comparable to an effect where the frequency of the previous stimuli is relevant. Similarly, a mechanistic explanation of the Weber principle (Pardo-Vazquez et al., 2019) may assume specific frequency of the stimuli (Brus et al., 2019). In numerical cognition, see a similar explanation for the task where participants have to indicate a numerosity on a line: The observed logarithmic distortion of the value location is not the result of the logarithmic representation assumed in the psychophysics-based ANS model, but the integration of the present stimulus and the past stimuli (Cicchini et al., 2014). In another numerical cognition example, it was proposed that the logarithmic number representation that was originally motivated by the psychophysical models could be derived only by relying on the frequencies of the number words (Piantadosi, 2016). It was also found that, in children, previous trials may influence the performance and the measured ANS acuity (Odic et al., 2014). In a recent study, it was proposed that estimation of visual sets depends on past experience of similar estimations (Brockbank et al., 2022). Overall, several previous findings suggest that preceding stimuli and the frequencies of the numbers may influence the processing in the actual nonsymbolic stimulus.

One of the open questions to specify the role of the frequency in nonsymbolic numerical processing is what the frequency distribution of nonsymbolic numerical stimuli is in a natural environment. Unfortunately, it is not straightforward to specify this frequency. While the frequency distribution of symbolic numbers is widely studied (e.g., Dehaene & Mehler, 1992; Piantadosi, 2014, 2016), it is much harder or currently even impossible to quantify the frequency distribution of nonsymbolic numerosities. (a) In some cases, it is supposed that the frequency distribution of the symbolic numbers could be assumed for nonsymbolic stimuli as well, or at least the frequency of the symbolic numerical stimuli is generalized to nonsymbolic stimuli without considering possible differences between these formats (Piantadosi, 2016; Testolin et al., 2020). (b) Analysis of specific visual stimuli showed that a similar distribution of values could be observed in nonsymbolic numerosity as in symbolic numbers (Testolin et al., 2020), although it is not known how closely the utilized stimulus set mimics the natural environment. (c) One may also argue that the frequency distribution is currently impossible to specify because this quantification would depend on the visual and numerosity models that would be able to specify the produced input for the numerosity system (e.g., Anobile et al., 2014; Burr & Ross, 2008; Dehaene & Changeux, 1993; Stoianov & Zorzi, 2012; Verguts & Fias, 2004). Until a widely accepted model emerges, it is impossible to specify what aspects of the visual environment should be considered to find the frequency distribution of nonsymbolic numerical information. (d) Finally, we note that in some models, the frequency distribution is less relevant because the model does not provide considerably different predictions depending on different frequency distributions (Testolin et al., 2020). To summarize, currently it is not known what frequency distributions of the nonsymbolic numerosity could be observed in the natural environment, although several viable possibilities assume non-uniform distributions.

More generally, some recent works found that statistical regularities in linguistic environment are similar to behavioral effects in symbolic and nonsymbolic number comparison (Ren & Libertus, 2024; Rinaldi et al., 2022). However, note that because the psychophysical ANS model and statistical models, such as the DSS, may have similar predictions (Krajcsi et al., 2016), the observed correlation between linguistic regularities and comparison performance may reflect either a statistical influence of the linguistic environment or an artifact of the correlating psychophysical process and statistical regularities.

In summary, while the ANS model assumes that frequency has no role in nonsymbolic number processing, some considerations hint that stimulus frequency may be an influencing factor in psychophysical stimulus processing, although the distribution of everyday nonsymbolic stimuli is hard to specify.

#### Theoretical and methodological implications of possible statistics-based effects

Based on the considerations so far, while the ANS model assumes that nonsymbolic number comparison performance is influenced only by the ratio of the stimuli, it is possible that the statistics of the stimuli may have an effect on the performance as well. This potential effect may have theoretical and methodological implications. If the statistics of the stimuli turn out to influence the performance, then a next critical question is (a) whether the statistics of the stimuli play inherent role in psychophysical processing or (b) whether the statistics-based effects are added to the regular psychophysical processing. (a) If the role is inherent, then it means that the ANS models or, more generally, the psychophysical models should be refined. Although psychophysical models seem to have solid foundations, many questions of that area are open and debated (Kingdom & Prins, 2010); for example, previous stimuli may fundamentally influence the stimulus processing (Petzschner et al., 2015). Similarly, while the ANS model has firm fundamentals (Dehaene, 2007), many essential and defining aspects of the psychophysical model are questioned and debated (Anobile et al., 2014; Burr & Ross, 2008; Cohen Kadosh & Walsh, 2009; Krajcsi et al., 2024a; Leibovich et al., 2017; Piantadosi, 2016; Stoianov & Zorzi, 2012). (b) Alternatively, if the statistics have an effect on the performance, but these effects are not inherent to the psychophysical component but they are added to the regular psychophysical processing, then these effects influence the measured behavior and the interpretation of the previously measured phenomena. Measuring the ANS sensitivity is essential in many fundamental theoretical questions, such as the development of numerical abilities or mathematical disabilities (Halberda et al., 2012; Piazza et al., 2010; Schneider et al., 2017). Still, the methods with which the ANS is measured may include unexpected pitfalls, and the practice of the measurement methods are disputed (Chesney, 2018; DeWind et al., 2015; Dietrich et al., 2015; Gebuis & Reynvoet, 2012; Krajcsi, 2020; Krajcsi et al., 2024a; Leibovich et al., 2017). If the possible statistical properties of the stimuli are influential and they are not controlled (e.g., adaptive techniques intentionally avoid uniform frequencies of the stimuli), they may introduce bias to the ANS sensitivity measurements. More generally, psychophysical measurements that ignore the possible effect of the statistical properties of the stimuli may be unnecessarily imprecise and/or biased. In addition, because previous models did not consider the potential influence of the stimulus statistics, the theories could not control for those influences in the observed phenomena, and the models may rely on biased data. For example, the studies contrasting the ANS and the DSS models rely on the assumption that the statistical properties cannot mask or overwrite the psychophysics-based phenomena in nonsymbolic number processing, but they can influence only the network-based symbolic number processing. If stimulus statistics have a considerable influence on performance with nonsymbolic numbers too, then the observed statistics-based effects in symbolic number comparison tasks (Krajcsi et al., 2022) could not be conclusive because the formerly observed statistics-dependent effects may not be exclusive to symbolic numbers; therefore, the results cannot demonstrate qualitative differences between symbolic and nonsymbolic number processing.

To summarize, it is possible that these statistical properties of the stimuli have effect on nonsymbolic number processing performance, which may question the current ANS model where the ratio effect is not influenced by the statistical properties of the stimuli, or that may question the measurements methods with which the ANS is quantified.

### The aims of the present studies

While previous works assumed that nonsymbolic number comparison is not influenced by the statistical properties of the stimuli, to our knowledge this has not been tested empirically. The potential effect of the statistical properties of the stimuli on nonsymbolic numerical comparison performance would change both the methods with which the ANS can be measured and the theories that rely on those collected data.

Here, we investigate (a) whether the nonsymbolic distance effect is influenced dominantly by the manipulation of the association between the values and the “larger” response, and (b) whether the nonsymbolic size effect is influenced by the frequency distribution of the values. A follow-up question is that if stimulus statistics influence the performance, then is the statistics-based effect an inherent part of the psychophysical processing (i.e., the statistics of the stimuli modify the psychophysical process itself) or is it an additional effect on the psychophysical processes?

Note that the present work does not contrast the ANS and the DSS models, since both the pure ANS and the hybrid ANS–DSS frameworks (Fig. 2) assume that nonsymbolic numbers are processed by the ANS which system is assumingly not sensitive to the statistical information of the stimuli. Instead, the present work tests a common assumption of the two models, namely that nonsymbolic number processing is not influenced by the statistical properties of the stimuli. Despite some other works raising the potential role of statistical information in nonsymbolic number processing, this role has not been tested empirically before.

## Study 1. The flexibility of the distance effect in nonsymbolic numerosity comparison

In the first study, we investigated whether the manipulation of the association between numerosities and small–large categories influence the distance effect in nonsymbolic numerosity comparison task. In symbolic notations (in artificial new symbols and in the Indo-Arabic notation), previous works have demonstrated that the association manipulation essentially modifies the distance effect (Kojouharova & Krajcsi, 2018; Krajcsi & Kojouharova, 2017).

### Methods

**Fig. 3:**
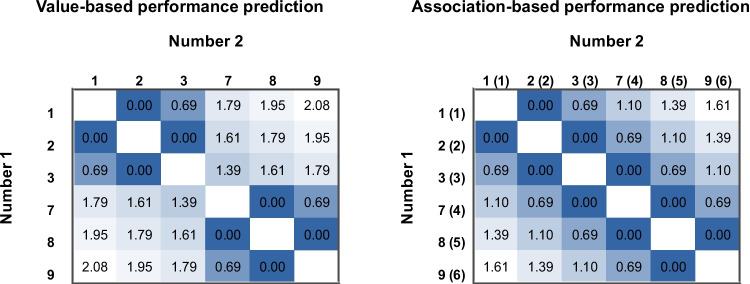
The distance effect regressors for the whole stimulus space used in the present study based on the value-based model (in line with the psychophysical models) and on the association-based model (prediction of the DSS model) are shown. The regressors were calculated as log(*large*–*small*), where log is the natural logarithm, *large* and *small* are the large and small numbers of the pair, and either the value of that number (in the value model) or the order of that number (in the association model, displayed in parentheses, e.g., 7 is in the 4th place of the ordered set; see also Table 1) was used. Note that to avoid the subitizing range (see the text for more details), in the study, the values were multiplied by 5 to get the numerosity of the to-be-presented arrays, but the predictions of the 1–9 or the 5–45 ranges in a regression analysis are the same: Since log(5×large – 5×small) equals log(5) + log(large – small), the best fit is shifted by log(5) when modifying the range from 1–9 to 5–45, but the goodness of fit, or the weight of the regressors should be the same. Note that expected performance also includes the size effect which is not displayed here for clarity (i.e., the present model includes only the distance effect, but not the size effect; see Fig. 1). Darker shades indicate worse performance

Participants indicated which of the two arrays of dots are more numerous. The numerosity of the arrays could be between 5 and 45 in steps of 5, excluding 20, 25, and 30. Omitting the middle sub-range of the whole range modifies the co-occurrences of the numerosities and small-large categories. The effect of this co-occurrences modification on the distance effect was measured. Co-occurrence can be the proportion of a number being smaller or larger in a comparison task (see Table 1). If performance is directed by the value (such as in the ANS model), the distance effect mainly depends on the distance of those values (which is a consequence of the ratio of the values, i.e., value-based performance in Fig. 3, left side). On the other hand, if the performance is mainly directed by the co-occurrences (such as with symbolic stimuli in the DSS model), the distance effect depends on the proportion of being smaller in a comparison task (which is the order of the value if the values are presented with equal probability, i.e., association-based performance; see an example in Table 1 and in Fig. 3, right side). In this test, the performance is measured in a single task, and two competing model predictions (i.e., the value-based and the association-based predictions) will be contrasted on that performance.

**Table 1:** The proportion of being smaller or larger in a comparison task when the values are presented with equal probability

Values	5	10	15		35	40	45
Proportion of being smaller in a comparison	100%	80%	60%		40%	20%	0%
Proportion of being larger in a comparison	0%	20%	40%		60%	80%	100%

#### Using the distance and size effect regressors in the analyses

Note that while the ANS model predicts a ratio effect in a comparison performance task (Fig. 1), this effect can be measured with either the distance or the size effects because the latter effects correlate with the ratio effect, and, in that model, they are influenced only by the ratio effect. In line with this assumption, many numerical cognition works (starting with the seminal work of Moyer & Landauer, 1967) measure the distance effect instead of the ratio effect since the distance effect is easier to measure and calculate. Similarly, in the present two studies, assumed ratio effect properties are approximated with the distance (Study 1) and size (Study 2) effects.

Additionally, in linear regressions, the use of the distance and size effects regressors instead of a ratio effect regressor allows us to measure the possible distance and size effect changes of the experimental manipulation more directly, and to investigate the possible dissociation between the distance and size effects. Thus, the present analysis methods focus on the specific phenomena the statistical properties of the stimuli modify in symbolic comparison tasks (Kojouharova & Krajcsi, 2018, 2019; Krajcsi & Kojouharova, 2017; Krajcsi et al., 2016).

Finally, in these studies, the size and distance effects can be investigated independently. In some versions of the distance and size effect models or regressors, the two regressors do not correlate. For example, when the distance is the difference between two values, the size is the sum of the values, and all possible number pairs of a value range are presented, the two regressors do not correlate. (This is also true, when the distance effect is calculated as the logarithm of the difference, such as in these two studies, but it is not true in some other cases, e.g., when the size is not the sum of the two values, but the power of the sum, such as in the illustration in Fig. 1.) Choosing such regressors for the analyses has several advantages. First, a potentially non-influential regressor will not be observed as an influential regressor, only because it correlates with an influential regressor. Second, simple linear regressions will lead to the same results as multiple linear regressions that use the regressors simultaneously.

#### Participants

Power analysis relied on previous results with symbolic number comparison tasks (Kojouharova & Krajcsi, 2018; Krajcsi & Kojouharova, 2017). Based on the logarithm model of reaction time data, in the first experiment of Krajcsi and Kojouharova (2017), for a paired Wilcoxon test, the effect size in *d*_z_ was −0.61, which requires 32 participants for 95% power (calculated with G*Power 3.1.9.6; Faul et al., 2007). Note that the replication experiment in that paper had a smaller effect size, while a similar study with Arabic numbers (Kojouharova & Krajcsi, 2018) had a larger effect size; therefore, the applied effect size can be considered representative of the present power analysis. Although the present sample size is somewhat lower than the power analysis-based 32 participants, sensitivity power analysis (the effect size that can be reached with 28 participants with 95% power) shows that still a 0.65 effect size can be demonstrated, and power-determination analysis (the power based on a −0.61 effect size and 28 participants) confirms that the present sample size is still appropriate for 92% power (while many studies ensure only 80% power; Giner-Sorolla et al., 2023).

Thirty-two university students participated in the study. The data of three participants were excluded from further analyses because they misunderstood the response key assignments or responded randomly. The data of another participant were excluded because the *R*^2^ of the distance effect fit for the reaction times (see details below) was close to 0, whereas for all other participants, this value was between 0.4 and 0.7. The data of 28 participants were analyzed (20 women, *M* = 21.3 years, *SD* = 2.5 years). No further demographic data were collected. The present study was carried out in accordance with the recommendations of the Department of Cognitive Psychology ethics committee (ELTE PPK 201410) and in accordance with the Declaration of Helsinki. All subjects gave written informed consent before the start of the study.

#### Stimuli and procedure

In a numerosity comparison task trial, two arrays of dots were presented on the two sides of the screen. The stimuli remained visible until response, and the participants had to choose the side with more dots by pressing one of the two response keys. The response was followed by an empty screen for 500 ms, then the next trial started.

The pairs consisted of all possible combinations of the numbers 5, 10, 15, 35, 40, and 45 (i.e., multiples of 5 between 5 and 45, excluding multiples between 20 and 30), excluding ties. The numerosity range did not include numbers smaller than five to avoid the subitizing range, that is, small numerosities that are enumerated relatively fast and with fewer errors (Kaufman et al., 1949) because the subitizing range is supposed to be processed by a pattern detection mechanism (Krajcsi et al., 2013; Mandler & Shebo, 1982) and not by the ANS (Revkin et al., 2008). All numerosity pairs were presented 20 times, resulting in a total of 600 trials. The order of the trials was randomized.

In an array of dots, black and white dots were shown against a gray background in random positions (Dakin et al., 2011); therefore, the luminance of the stimuli was not informative about the numerosity. Dots of an array were drawn randomly in a 2 × 2° area, with a dot diameter of 0.2°; therefore, dot size was not correlated with the numerosity, while density and convex hull correlated with the numerosity. While, in these stimuli, perceptual nonnumerical features are not controlled entirely, controlling for the nonnumerical features is impossible (DeWind et al., 2015). More importantly, the perceptual features that may influence the response are processed by the same kind of psychophysical processes; therefore, it is reasonable to assume that the association statistics may influence those nonnumerical but psychophysical properties as well.

The generation and presentation of the stimuli and recording of the responses were managed by the PsychoPy software (Peirce, 2007). A session lasted approximately 20 min.

#### Transparency and openness

We report how we determined our sample size, all data exclusions, all manipulations, and all measures in the study. All raw data are available online (https://osf.io/5rzte/). Data were analyzed using *LibreOffice Calc* (Version 7.1), *CogStat* (Version 2.1; Krajcsi, 2021), *jamovi* (Version 2.3.26; The Jamovi Project, 2020), and *pyddm* (Shinn et al., 2020). This study’s design and its analysis were not preregistered.

### Results and discussion

**Fig. 4:**
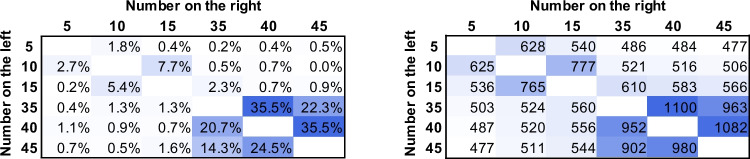
Group mean error rate (left) and reaction time in ms (right) for all numerosity pairs

Mean error rate and median reaction time (both correct and erroneous trials were included, since, in psychophysical tasks, erroneous responses may rely on the same mechanisms as correct responses; extremely slow responses were not removed because the median is robust to slow outliers) for each number pair and each participant were calculated. Means across participants for both error rate and reaction time are shown in Fig. 4 (the average error rate was 6.2%, and the average reaction time was 561 ms). Short response latencies demonstrate that the participants used estimation to solve the task because the responses were too fast to use the counting strategy. Visual inspection suggests that the behavioral response pattern (Fig. 4) followed the value-based model (left of Fig. 3), while the behavioral response patterns observed in previous studies with symbolic comparison tasks (Kojouharova & Krajcsi, 2018; Krajcsi & Kojouharova, 2017) followed the association-based model (right of Fig. 3). Note again that the measured patterns are somewhat different compared to the performance prediction seen in the left panel of Fig. 3 because the prediction included only the distance effect but not the size effect (see Fig. 1)—that is, in the measured data, performance with large numbers (i.e., bottom right part of the displayed stimulus space) is more difficult than with small numbers (i.e., top left part of the displayed stimulus space). Also note that since the logarithmic distance effect and a possible sum-based size effect regressors do not correlate here (see above in Methods), adding the size effect regressor to the present analysis would not modify the findings about the weights of the distance effect regressor.

This observation was confirmed by the statistical analyses. Fitting the value-based (logarithm of the distance of the values; left panel of Fig. 3) and the association-based (logarithm of the distance of the values’ orders; right panel of Fig. 3) models^3^ on the group average results, the value-based model fit was higher than the association-based model fit both for error rates (*R*^2^ =.44 for value-based model, and *R*^2^ = 0.28 for the association-based model), and for reaction time (*R*^2^ =.62 and *R*^2^ =.44). Note that, here, as a neutral choice in terms of theories, the distance effect was fitted, and not the ratio effect as predicted by the ANS (similar to many other works, such as in the seminal Moyer & Landauer, 1967, paper). Note again that *R*^2^ values cannot be close to 1 because, for example, the size effect was not included in the model which effect is known to influence the performance.

In a parallel analysis, the two models were fitted to the individual participants’ data (mean error rate and median reaction time were calculated for each number pair in each participant), and the *R*^2^ values were calculated for each participant. These *R*^2^ values were compared as repeated measures ordinal variables. This analysis confirms the previous results: The value-based model fits better than the association-based model both for error rates (median *R*^2^ values are.34 and.21, respectively, and the difference is highly significant, *T* = 0, *p* <.001, tested with paired Wilcoxon test, where *R*^2^ values are handled as ordinal variables) and for reaction times (median *R*^2^ values are.53 and.39, *T* = 0, *p* <.001).

Overall, it was found that, unlike in the symbolic number comparison task (Kojouharova & Krajcsi, 2018; Krajcsi & Kojouharova, 2017), in nonsymbolic numerosity comparison task, the distance effect was mainly influenced by the values of the stimuli and not their association with the “larger” response. In terms of hypothesis tests of the symbolic comparison task, the value-based model was significantly worse than the association-based model (Kojouharova & Krajcsi, 2018; Krajcsi & Kojouharova, 2017), while in the nonsymbolic comparison task, the value-based model was significantly better than the association-based model. Therefore, distance effects in symbolic and nonsymbolic comparison tasks differ markedly: While, in nonsymbolic comparison, the distance effect is dominantly rooted in the ratio effect and not in the value/large–small associations (in line with the ANS model, including both the pure ANS framework and the hybrid ANS–DSS framework), in symbolic comparison, the distance effect is dominantly the consequence of the value/large–small associations and not a psychophysical process (in line with the DSS model, and the hybrid ANS–DSS framework).

## Study 2. The flexibility of the size effect in nonsymbolic numerosity comparison

In the second study, we investigated whether the manipulation of the frequency of the values influences the size effect in a nonsymbolic numerosity comparison task. In symbolic notations (in artificial new symbols and in Indo-Arabic notation), previous studies have demonstrated that the frequency manipulation modifies the size effect (Kojouharova & Krajcsi, 2019; Krajcsi et al., 2016).

### Methods

**Fig. 5:**
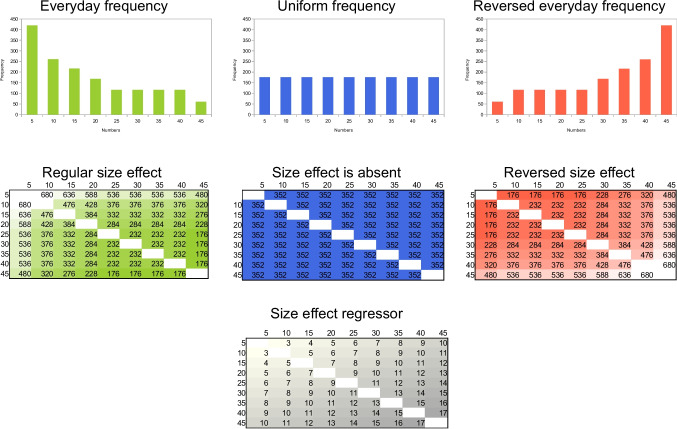
**Top**. Frequencies with distribution similar to everyday life, uniform distribution and a distribution of reversed everyday life, reflecting the power-based observed frequencies of symbolic numbers in everyday life (Dehaene & Mehler, 1992). **Middle**. Possible expected size effect in the three frequency conditions on an arbitrary scale if the frequency entirely influences performance. Expected performance for a number pair was calculated as the sum of the frequencies of the values. Darker shade indicates worse performance. **Bottom**. The size effect regressor that was used to detect the size effect changes, computed as the sum of the two initial numbers (i.e., the presented value divided by five). Darker shade indicates worse expected performance (note again that on an arbitrary scale, the more difficult task could be either larger or smaller value). (Color figure online)

Following the logic and paradigm of similar studies with symbolic number comparison (Kojouharova & Krajcsi, 2019; Krajcsi et al., 2016), in a nonsymbolic comparison task, participants decided which of two concurrently observable arrays of dots include more dots. In three conditions, the numbers were presented according to the everyday symbolic number frequencies (i.e., smaller numbers are presented more frequently than larger numbers), according to the reverse of the everyday number frequencies (i.e., larger numbers are presented more frequently than smaller numbers), and uniformly (i.e., all numbers are presented with equal frequencies; see the top panel of Fig. 5). If performance follows mainly the frequency manipulation, respectively, a regular, a reversed, or no size effect (middle of Fig. 5) should be observed in the data. (Note that these predictions do not include the distance effect, see Fig. 1 for an example on how the distance and size effects differ and how they can be combined.) Unlike in the previous study, where a single condition task was used and two model predictions were contrasted, here, three conditions are used and a single effect is measured to compare the performance in the three conditions. In other words, while the analysis of the previous study contrasted the models, the present analysis contrasts the conditions.

#### Participants

For a power analysis, the results of similar symbolic comparison tasks were used (Kojouharova & Krajcsi, 2019; Krajcsi et al., 2016). For a conservative estimation, the results of the study with Indo-Arabic stimuli were used (Kojouharova & Krajcsi, 2019) where the effect size was smaller compared with the study with new symbols (Krajcsi et al., 2016). Based on the reported analysis of variance (ANOVA) results on error rates, the effect size in f was 0.64, which requires 42 participants for 95% power (calculated with G*Power 3.1.9.6; Faul et al., 2007).

The data of 68 participants were collected. The data of seven participants were excluded (the session was not completed, the participant gave random responses, they mixed up the response buttons, or the size effect slope was an extreme outlier with 6.7 *z*-score). The data of 61 participants (mean age = 21.2 years, *SD* = 2.08, 19 men; no further demographic data were collected) were analyzed, where 16, 24, and 21 participants were in the everyday, uniform, and reversed everyday conditions, respectively.

#### Stimuli and procedure

Pairs of dot arrays were presented to the participants in a number comparison task. In each trial, the participants decided which side had more dots by pressing the R and I keys of a keyboard. The two arrays remained visible until response.

Slightly different methods were used for choosing the stimuli in the three frequency conditions. (1) Following the methods of Kojouharova and Krajcsi (2019), in the everyday frequency condition, the base frequency of each number was calculated according to the frequency_value_ = value^−1^ × 10 formula which yielded the following (rounded) frequencies (value:frequency): 5:10, 10:5, 15:4, 20:3, 25:2, 30:2, 35:2, 40:2, 45:1 (see Dehaene & Mehler, 1992; Krajcsi et al., 2016). (As discussed in the Introduction, it is not known if the frequency distribution of nonsymbolic numerosity follows the frequency distribution of the symbolic numbers. Still, any frequency distribution could be appropriate to investigate the effect of frequency on number processing.) The two permutations of all numbers excluding ties were generated, resulting in 794 trials. Each pair was presented twice for a total of 1,588 trials. The frequencies of the specific values are displayed in the left of the top row of Fig. 5 (note that these are the frequencies of the numbers resulting from the permutations, and not the number pairs or the base number frequency from which the number pairs were generated). The most frequent number pairs (i.e., 5 vs. 10, and 10 vs. 5) were presented 100 times, and the least frequent pairs (e.g., 40 vs. 45) were presented four times. (2) The same procedure was repeated for the reversed everyday frequency condition, but the frequencies were reversed before creating the number pairs(i.e., value:frequency: 5:1, 10:2, 15:2, 20:2, 25:2, 30:3, 35:4, 40:5, 45:10; right of Fig. 5, top row). (3) In the uniform frequency condition, all possible pairs excluding ties were generated (middle of Fig. 5, top row). Each pair was presented 22 times, resulting in 1,584 trials overall.

Similar to Study 1, sets with less than 5 objects were avoided, and numbers described above between 1 and 9 were multiplied by 5, resulting in a number range between 5 and 45. The arrays of dots were displayed with the same perceptual properties as in Study 1.

The generation and presentation of the stimuli and recording of the responses were managed by the PsychoPy software (Peirce, 2007). A session lasted approximately 30 min.

### Results and discussion

The raw data are available online (https://osf.io/5rzte/).

#### Changes in the size effect

**Fig. 6:**
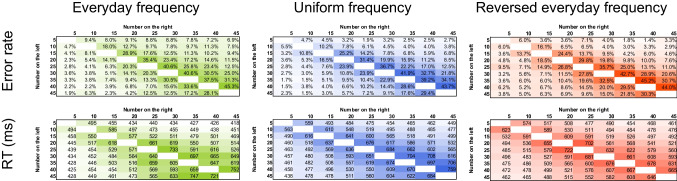
Group error rate and reaction time for all number pairs in the three frequency condition groups. (Color figure online)

**Fig. 7:**
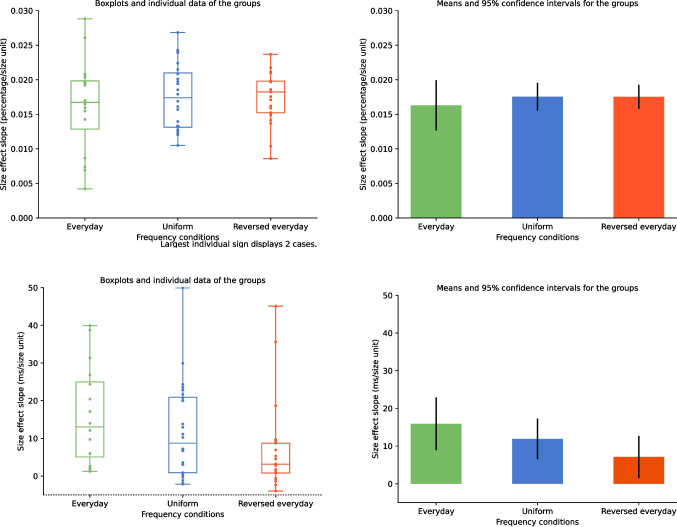
Box plot (**left**) and mean estimations (point estimation and 95% CI; **right**) of the slope of the size effect regressor for the error rate slopes (**top**) and reaction time slopes (**bottom**) in the three frequency conditions. (Color figure online)

Mean error rate and median reaction time (both correct and erroneous trials were included) were calculated for each number pair and for each participant. The mean values across participants are displayed in Fig. 6 (the average error rate was 17.3%, and the average reaction time was 519 ms). Like in the first study, fast responses demonstrate that the participants used estimation to solve the task instead of the counting strategy. Visual inspection of the performance in the three conditions shows that value is the leading factor to form behavioral performance, that is, the ratio effect is visible in all conditions (Fig. 6; see Fig. 1 for how the ratio effect looks like in this visualization). However, a more detailed analysis shows that size effect changes with the frequency manipulation. The size effect was calculated for each participant as the slope of the size regressor fitted on the mean error rate and median reaction time data considering each number pair as a separate data point (Fig. 6). The size effect regressor value for a number pair was the sum of the values, as seen in the bottom of Fig. 5. Note that this regressor only approximates the predicted size effects seen in middle row of Fig. 5, but the regressor is neutral in the sense that any predicted effect sizes can be detected, where the value of the slope can differentiate between the three characteristic expected patterns: Depending on the observed pattern, the slope should be positive, zero, or negative. Another advantage of using the sum of the numbers as the size effect regressor is that since the distance and size effect regressors do not correlate (see above in the Methods section of Study 1), adding the distance effect regressor would not modify the findings about the weight of the size effect regressor. Following this method, each participant had two size effect slopes: One for the error rate patterns and one for the reaction time. The size effect slopes were compared between the three conditions (Fig. 7): While the difference was not significant in error rates (one-way ANOVA), *F*(2, 58) = 0.35, *p* =.704, ω^2^ = −0.022, it was significant in reaction time (Kruskal–Wallis test, since the normality assumption was violated), χ^2^(2, *N* = 61) = 6.49, *p* =.039, ω^2^ = 0.039, where post hoc Dunn’s test shows a significant difference between the everyday and reversed everyday conditions (*p* =.012).

The present results show that nonsymbolic numerical comparison performance is partly influenced by the frequency of the stimuli: While the frequency of the stimuli changes the size effect to the expected direction (size effect slope is largest for the everyday condition, and smallest for the reversed everyday condition), the frequency of the stimuli in the session is not the only influence (the slope for the reversed everyday condition is not negative, and the slope for the uniform condition is not zero). One main question is whether this effect is an additional effect on top of the psychophysical ratio effect or an inherent component of the ratio effect.

#### Possible changes in the distance effect

One way to explore whether the size effect change reflects inherent change in the psychophysical process or whether it is an additional effect is to investigate whether the distance effect changed together with the size effect. In the psychophysics-based ANS model, the distance effect and the size effect are two ways to measure the single ratio effect (Fig. 1). In other words, it is the ratio effect that is generated, however, when one measures the distance or the size effects, these latter effects will be observable because they correlate with the ratio effect. Importantly, in the ANS model, only the sensitivity of the representation, termed the Weber fraction, is responsible for the performance (Dehaene, 2007; Krajcsi et al., 2018). If the frequency of the stimuli changed the psychophysical process, it can be only the Weber fraction that mediated this modification, therefore, the whole ratio effect should be changed. Consequently, not only the size effect but also the distance effect should be changed because those two effects stem from purely on the ratio effect. Moreover, according to the ANS model and a potential ANS-based ratio effect change, the effect size of the distance effect and its change should be similar to the effect size of the size effect and its change (Fig. 1, bottom row); therefore, the power of the relevant distance effect hypothesis test should be similar to the power of the size effect hypothesis test observed above. To summarize, the ANS model predicts that if the frequency of the stimuli modifies the ANS processing inherently, then the Weber fraction along with the ratio effect should be changed, and, consequently, the distance effect should be modified too.

Similar to the size effect analysis, the three conditions were compared, however, not the size effect regressor (bottom of Fig. 5), but a distance effect regressor was used. Similar to Study 1, the distance was calculated as the logarithm of the difference of the values. The slope of the distance effect regressor was calculated in each condition; therefore, each participant had a single distance effect slope, which was compared across the three frequency groups. (Like in the case of the previous analysis, since the distance and size effect regressors do not correlate, adding the size effect regressor to the present analysis would not change the findings about the distance effect.) The difference was not significant either for the error rates (one-way ANOVA), *F*(2, 58) = 1.13, *p* =.331, ω^2^ = 0.004, or for the reaction time (Kruskal–Wallis test), χ^2^(2, *N* = 61) = 0.28, *p* =.867, ω^2^ = −0.032. These results mean that it is not the psychophysics-based ANS that mediates the change in the performance (at least not a classic form of the ANS), but an additional mechanism. In other words, because frequency manipulation of the stimuli influenced only the size effect but not the distance effect, it shows that it was not the psychophysics-based ratio effect that was influenced, but another mechanism should be responsible for the size effect change.

#### Diffusion model analysis

To explore the frequency-induced size effect further, diffusion model analysis was performed. Diffusion models assume that, in a decision process, evidence is collected until a decision threshold is reached (Ratcliff & McKoon, 2008; Ratcliff et al., 2016; Smith & Ratcliff, 2004; Voss et al., 2013). In a classic diffusion model, responses depend on four key parameters: drift rate, threshold, starting point, and nondecision time (Ratcliff et al., 2016; Shinn et al., 2020). Drift rate is the quality of the information the decision process relies on: Higher drift rate leads to faster and more precise responses. Threshold is the criterion for making the decision. Threshold is related to the speed–accuracy trade-off: Higher threshold leads to slower but more accurate responses. Starting point is the initial value of the evidence accumulator. Usually, it is midway between the two thresholds belonging to the two responses; however, it may be biased towards one of the thresholds (i.e., one of the responses), for example, because of the expected higher chance of one of the responses. Nondecision time is the time period that does not belong to the core decision process (e.g., early perceptual processing, motor response execution). Diffusion model parameters can be recovered from the behavioral data. Recovery is useful in behavioral studies because it can identify various sources of the responses (i.e., the parameters, such as the drift rate) behind the error rate and reaction time patterns (Ratcliff & McKoon, 2008; Ratcliff et al., 2016). Furthermore, it can resolve apparent contradictions where an effect is not visible in both the error rates and the reaction time (Wagenmakers et al., 2007), as in the present case, where the frequency-based size effect is observable in the reaction time data but not in the error rates.

In terms of diffusion models, psychophysical models assume that the ratio effect is the result of the task difficulty reflected in the drift rate (Palmer et al., 2005). Similarly, the ANS model assumes that the ratio effect is mediated by the drift rate (Dehaene, 2007), and this assumption has been confirmed by empirical measurements (Dehaene, 2007; Krajcsi et al., 2018). Therefore, one starting point of the present analysis is that if the frequency manipulation influences the core ANS process, then it is the drift rate that should be changed. On the other hand, a possible drift rate change does not necessarily mean that the ANS process is involved in the frequency-based changes because it is possible that an additional representation which contributes to the decision process which additional representation is responsible for the performance modification throughout the drift rate change.

In an initial analysis of the present data, the parameter recovery was performed with the EZ method (Wagenmakers et al., 2007) and also with the *pyddm* package (Shinn et al., 2020). The results showed that the size effect change is mediated by the threshold and nondecision time parameters, but not by the drift rate parameter (see the Supplementary Material). However, this result may be partially or entirely biased. The unequal number of trials between the cells of the design that is essential in the present experimental manipulation may introduce a bias in these recovery analyses. Alternatively, when some trials are removed to make the size of the cells equal, the number of trials becomes too low to provide reliable results. Additionally, the results suggest that the thresholds are changed within the trials, while the classic parameter recovery methods are not suitable to detect such changes. (See additional technical details about the analyses and the related issues in the Supplementary Material.) Therefore, a quantitative parameter recovery was impossible to perform on the present data. However, changes in the diffusion model parameters have characteristic effects on the error rate and reaction time patterns that can be used to reason qualitatively about the possible sources of the frequency-based size effect modification (Ratcliff et al., 2016).

#### Qualitative diffusion model parameter recovery

Different diffusion parameters have different effects on the response distribution—that is why it is possible to recover those parameters (e.g., see Ratcliff et al., 2016; or see the interactive demonstration in Alexandrowicz, 2020). Drift rate change is associated with relatively large changes both in the error rates and in the reaction time, and when the drift rate is increased, responses will be more precise and faster (Table 2). The threshold change is associated with large changes only in reaction time, but small change in error rates, and when the threshold is increased, responses will be more precise, but slower. That is, increasing both the drift rate and the threshold makes the responses more precise, but larger drift rate makes the responses faster, whereas larger threshold makes the responses slower. Note also, that the change of the error rates is smaller when the threshold is changed than when the drift rate is changed. Nondecision time change is associated only with reaction time change (Table 2). Because the participants may not have expectations about the response of the upcoming trials in our study, the starting point bias is unlikely to influence the decision.^4^

**Table 2:** The effect of some diffusion model parameters on response time and response precision

Effect on responses	Effect on the reaction time		Effect on the precision	
Change in the diffusion parameter				
Increased drift rate	Faster responses	↓	More precise responses	↑
Increased threshold	Slower responses	↑	Slightly more precise responses	↑
Increased nondecision time	Slower responses	↑	No change in precision	○

Comparing the present result with the prediction of the diffusion model described above, the lack of observable error rate change in the present results (both in the slopes and in the error rates directly; Figs. 6 and 7) is not in line with a possible drift rate change, but it is more probably a result of a threshold and/or nondecision time change (Table 2). Specifically, in the present results, more frequent values are processed faster (as observed in the direction of slope change resulting from the frequency manipulation). Here, a drift rate-based change (with larger drift rate for more frequent stimuli) would predict that the error rate of more frequent values should be smaller and the effect should be relatively large, while a threshold-based change (with smaller threshold for more frequent stimuli) predicts that the error rate of the more frequent values should be larger, although that change in the error rates should be relatively small. Descriptive data show a pattern that is in line with the latter possibility (Fig. 7): In the reversed everyday condition, the error rates slope is higher than in the everyday condition, which means that more frequent stimuli have higher error rates. At the same time, the frequency-based difference in the error rate is not significant, which can also be compatible with the smaller effect size prediction of the threshold change. (Note that the other potential source of the nonsignificant result is the lack of the size effect change, which we consider below.) Therefore, the data may suggest that, in nonsymbolic comparison, more frequent stimuli are processed with a smaller decision threshold. Finally, it must be mentioned that the reaction time change could be rooted in the change of the nondecision time as well. Nondecision time predicts that the error rate should not change, and the present results may be in line with this prediction: The observed error rate differences may be noise, and the error rate-based size effect change may be nonsignificant because of the true absence of the effect.

To summarize, the response pattern (significant effect on reaction time, nonsignificant effect on error rate, faster but more erroneous responses for frequent stimuli) is in line with the idea that for more frequent values the threshold and/or the nondecision time decreased, while the drift rate was not affected.

Note that while quantitative parameter recovery was not possible with the current data, qualitative analysis still could be informative about what diffusion parameters are responsible for some observed behavioral effects. A qualitative analysis is not less appropriate or more biased than a quantitative analysis; The main difference is that while a quantitative analysis provides numerical estimates of the diffusion parameters, a qualitative analysis provides only the presence and direction of the parameter changes.

The finding that frequency manipulation-based response change is not likely to come from drift rate change means that this performance change is not rooted in the psychophysical processes or in the ANS, but it is an additional mechanism, because the ANS model assumes that the psychophysical process is mediated by the drift rate. We do not assume that nonsymbolic comparison is supported by the DSS either because nonsymbolic comparison considerably differs from symbolic comparison. For example, in the present study, unlike in symbolic comparison, the distance effect was not influenced dominantly by the association of the values and the “larger” responses. Similarly, unlike in symbolic comparison, in nonsymbolic comparison, the size effect change is not mediated by the drift rate (see the next subsection). More generally, because most properties of the nonsymbolic comparison are in line with the ANS model and differs from symbolic comparison (see a critical review of these properties in Krajcsi et al., 2022), it is reasonable to assume that nonsymbolic comparison is supported by the ANS, while the frequency-based size effect modification is caused by an extra mechanism but not by the DSS.

Currently, it is hard to tell what mechanism could be behind this threshold change. It is probably not related to the speed–accuracy trade-off (e.g., Ratcliff et al., 2016) because instruction-induced speed–accuracy setting (i.e., participants set higher threshold when accuracy is stressed in the instruction, while they set lower threshold when speed is emphasized) is set before the trial, however, the frequency of the stimuli are not known before the trial in the present paradigm. In general, within-trial change of the threshold could be related to several properties of the stimuli, such as reliability of the input (Deneve, 2012), increasing urgency, or delay in feedback (Evans & Hawkins, 2019), however, it is not clear what property is critical in the frequency of the stimuli. Still, one possible hypothesis is that, after participants evaluate the frequency of the stimulus, they decrease the threshold for frequent stimuli because they estimate that frequently observed tasks can easily be solved by retrieval or other fast solutions and increased concern (i.e., increased threshold) is not needed. Future studies may investigate this hypothesis further.

#### Contrasting symbolic and nonsymbolic comparisons

Importantly, while, in the nonsymbolic numerical comparison task, the frequency manipulation-based performance change may not be rooted in the drift rate change, in the symbolic comparison task, the seemingly similar performance change is at least partly attributable to the drift rate change (Table 3). In a study that is similar to the present one, the frequency of the Indo-Arabic numbers was manipulated to form everyday, uniform, and reversed everyday frequency distributions (Kojouharova & Krajcsi, 2019). It was found that frequency conditions significantly influence not only the reaction time, but also the error rates. In addition, more frequent numbers were not only faster to process, but also less error-prone. Both the involvement of the error rate and the direction of its change suggest that the frequency-based behavior change was the result of the drift rate change (Tables 2 and 3). (Note that this cited work also reported the drift rate; however, the methodological constraints of differing trial numbers discussed above apply to those analyses too; therefore, those drift rate results may be biased as well.) In another study, frequency was manipulated to form a uniform and everyday frequency distribution for new symbolic notation (Krajcsi et al., 2016). It was found that the size effect was dominantly led by the frequency manipulation: In the uniform frequency distribution condition, the size effect slopes did not differ significantly from zero either for error rates or for reaction time, however, in the everyday frequency distribution condition, the size effect was significantly different from zero and from the previous condition in both error rates and reaction time. Critically, more frequent stimuli were faster and less error prone. Again, these data support the notion that, in a symbolic comparison task, the frequency manipulation affects the drift rate.

**Table 3:**
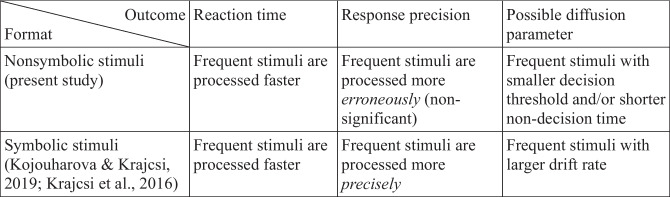
Change of behavior performance depending on the frequency of the stimuli and its possible diffusion parameter sources in nonsymbolic and symbolic comparison tasks

This contrast highlights that while the behavioral performance (or at least the reaction time) change suggests a similar pattern behind stimulus frequency-based change in nonsymbolic and symbolic number processing, diffusion model analysis identifies different mechanisms. More specifically, in line with the hybrid ANS–DSS framework, while nonsymbolic number comparison is in line with the psychophysical model (the ratio effect is rooted in the drift rate, while stimulus frequency can change the threshold and/or the nondecision time), symbolic number comparison relies on other mechanisms, the DSS (stimulus frequency change can modify the drift rate, which phenomenon is incompatible with the current psychophysical ANS model).

#### Changes in the size effect throughout the session

**Fig. 8:**
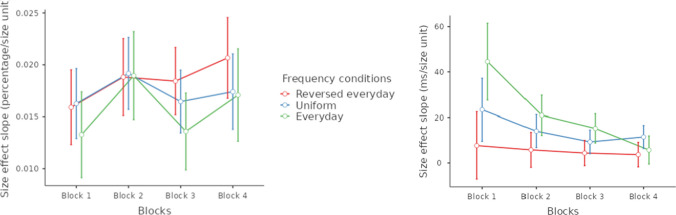
Size effect slopes in error rates (left) and reaction time (right) as a function of blocks (*x*-axes) and frequency conditions (lines). Error bars represent 95% confidence intervals. (Color figure online)

Since the stimulus frequency influenced the size effect in nonsymbolic comparison, we explored the change of this size effect modification throughout the session. The whole session was divided into four blocks, and the size effect slope was calculated for each block separately (Fig. 8). While, in the error rate slopes, no systematic change was observed, in the reaction time slopes, the influence of the stimulus frequency on the size effect is largest in the first block, and it decreases throughout the session. Two 4 (blocks as within-subject factor) × 3 (frequency conditions as between-subject factors) ANOVAs for the error rates and reaction time confirmed this observation. For the error rate, only the blocks had significant effect on the slopes, *F*(3, 174) = 3.370, *p* =.02; *F*(6, 174) = 0.569, *p* = 0.75; and *F*(2, 58) = 1.36, *p* =.27 for the block main effect, the frequency main effect, and the interaction, respectively; where post hoc Tukey test showed that the second block had higher error rate slopes than the first and third blocks (*p*s <.047). For the reaction time, all effects were significant: *F*(3, 174) = 11.72, *p* <.001; *F*(6, 174) = 3.28, *p* = 0.004; and *F*(2, 58) = 6.21, *p* =.004 for the block main effect, the frequency main effect, and the interaction, respectively. The significant interaction shows that the influence of the stimulus frequency decreased over time. While, in the first block, the confidence intervals do not overlap, noting significant differences between the three groups, this difference disappears by the fourth block, as seen in the overlapping confidence intervals.

This pattern of decreasing influence of the stimulus frequency in nonsymbolic comparison is in a clear contrast with a stable influence of stimulus frequency in symbolic comparison: In a symbolic number comparison task, the size effect is stable across the blocks of a session both with artificial new symbols and with Arabic numbers (Kojouharova & Krajcsi, 2019; Krajcsi & Kojouharova, 2023). While we have no available explanation for why the supporting processes produce different size effect changes, this difference indicates again that symbolic and nonsymbolic numbers are processed differently, and the size effect modifications that are induced by the stimulus frequency are supported by different generators in different stimulus formats.

To summarize, the second study of the present work revealed that the stimulus frequency influences the size effect in nonsymbolic comparison. However, this modification may not be rooted in the psychophysical process, but it is an additional mechanism because (a) the distance effect is not modified (whereas the psychophysical ANS model assumes the concurrent changes of the distance and size effects), and (b), in terms of diffusion models, the size effect change is mediated by the threshold and/or the nondecision time but not by the drift rate (whereas the ANS model assumes that the drift rate is the mediator). In addition, the size effect change may not be rooted in the DSS either, since (a) DSS-based symbolic comparison is rooted in drift rate changes, while the present nonsymbolic size effect change is rooted in the threshold and/or the nondecision time, and (b) the influence of the stimulus frequency is stable throughout the session in symbolic comparison, whereas it decreases throughout the session in nonsymbolic comparison.

## General discussion

The present two studies investigated (a) the role of the associations between the values and the “larger” response and (b) the role of the frequency of the stimuli in nonsymbolic number comparison task. These factors have been found to be essential in symbolic number comparison (Kojouharova & Krajcsi, 2018, 2019; Krajcsi & Kojouharova, 2017; Krajcsi et al., 2016). While neither the ANS nor the DSS model assumes statistical influence in nonsymbolic number comparison task (Dehaene, 2007; Krajcsi et al., 2022), some other results hint that such effect is possible (Brockbank et al., 2022; Brus et al., 2019; Cicchini et al., 2014; Odic et al., 2014; Petzschner et al., 2015; Piantadosi, 2016).

It was found that, while the association between the values and the “larger” response does not have a fundamental effect (statistically, a relatively large effect size) on the responses and on the distance effect, the frequency of the stimuli affects the size effect. Importantly, the latter frequency-based size effect is rooted in an additional mechanism beyond the psychophysical mechanism: (a) the frequency manipulation did not modify the distance effect (whereas the psychophysical account assumes the concurrent modification of both the size and distance effects), and (b) qualitative diffusion model parameter recovery showed that the frequency manipulation-induced size effect changes were mediated by the threshold and/or the nondecision time change (whereas psychophysical processes are mediated by the drift rate). Finally, it was found that the influence of the stimulus frequency on the size effect is decreasing throughout the session in nonsymbolic comparison.^5^

These results highlight again that nonsymbolic and symbolic number comparisons are processed fundamentally differently and are not backed by similar mechanisms. First, while, in the case of the symbolic comparison distance effect, the association between the values and the “larger” response is dominant, the role of this association was not observed in the current nonsymbolic comparison task. Second, although stimulus frequency manipulation can modify the size effect in both symbolic and nonsymbolic number comparison, this size effect change is mediated by the drift rate only in symbolic comparison but not in nonsymbolic comparison. Third, the influence of the frequency manipulation is decreasing throughout the session in nonsymbolic comparison, whereas it remains relatively stable in symbolic comparison. All of these differences are in line with the hybrid ANS–DSS framework (Fig. 2), which proposes that nonsymbolic number comparison obeys the Weber principle, whereas symbolic number comparison relies on a qualitatively differing representation mainly influenced by the statistical properties of the stimuli.

These results also reveal important details about the heterogeneous sources of the numerical comparison size effect. In the literature that supports the pure ANS framework, the comparison size effect is considered as another measurement of the psychophysical mechanism-based ratio effect. However, the present results together with previous findings suggest that at least three mechanisms may be responsible for generating the size effect (Table 4). (1) In line with the ANS model, a component of the observed size effect is rooted in the psychophysics-based ratio effect, which is mediated by the drift rate. This is the mechanism that the majority of the numerical cognition literature assumes to be the main or only source of the size effect. (2) A next source of the observed size effect is the modification of the decision threshold and/or the nondecision time depending on the stimulus frequencies. This is the mechanism the present work revealed. (3) Another source of the size effect depending on the stimulus frequency is a drift rate-mediated mechanism that is observable in symbolic number comparison and which does not obey the Weber principle (Kojouharova & Krajcsi, 2019; Krajcsi et al., 2016). In terms of these size effect generators, symbolic and nonsymbolic comparisons rely characteristically differently on these mechanisms. In nonsymbolic comparison, sources (1) and (2) are probable candidates for the size effect. However, in symbolic comparison, at least source (3) is responsible for the size effect, but the role of source (2) or the potentially marginal role of source (1) is currently unknown. While it has been demonstrated that symbolic number comparison performance is mainly led by the statistical properties of the stimuli (Krajcsi et al., 2022), it is possible that the ANS may have a partial role. In a recent study, the stimulus frequency was manipulated in a new artificial number notation to test the possible partial role of the ANS in symbolic number comparison (Krajcsi & Kojouharova, 2023). None of the novel phenomena that were described could demonstrate the role of the ANS. Therefore, even if source (1) has an effect in symbolic number comparison, its role cannot be dominant and its possible role is yet to be demonstrated. The multiple sources of the size effect are compatible with a parallel finding of the distance effect in symbolic comparison, where signal detection theory-based analysis demonstrated that the distance effect is lead not only by the sensitivity index, but also by the bias index (Hepdarcan et al., 2021). Overall, the present results together with previous findings paint a rich picture of the seemingly simple size effect.

**Table 4:**
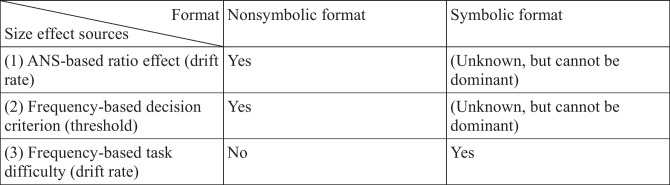
Different sources of the numerical comparison size effect in nonsymbolic and symbolic formats

One may raise that the effect of the experimental manipulation of the stimulus statistics in these studies may interact with the effect of previous and, importantly, unknown stimulus statistics, whereas the observed behavioral change does not reflect the experimental stimulus statistics manipulation; therefore, the current conclusions are not strongly justified. Here, an important aspect to consider is how new statistical information is integrated into the perceived former stimulus statistics. We assume that the new statistical pattern has a nonlinear monotonic effect on behavior, that is, more frequent stimuli or stronger conditional frequencies make responses easier, although it is unspecified how easier the responses can be. With this reasonable assumption, the suggested interaction is not possible. Unless empirical data or reasonable theoretical considerations suggest alternative assumptions, the current interpretation of statistical effects is the parsimonious solution.

As a methodological implication, the effect of the frequency on the response performance may affect any psychophysical measurement (such as the measurement of the Weber fraction) when the frequency of the stimuli are not equal. For example, in adaptive measurement methods, the stimuli around the psychophysical threshold are presented more frequently than the stimuli around the ceiling or floor performance. Theoretically, this may influence the performance. This potential bias may be a concern not only in numerical cognition (Dietrich et al., 2015), but also in any psychophysical measurements. However, it is important to highlight that psychophysical measurements often rely on error rates (Palmer et al., 2005; Pardo-Vazquez et al., 2019), which are less influenced by the stimulus frequency than the reaction time data. Additionally, it is not known how strong that effect could be in those circumstances. Overall, while the frequency of the stimuli may influence psychophysical measurements, it is not clear yet whether this influence causes considerable bias in those measurements.

While, as described in the motivation of the present work, neither the ANS nor the DSS models assumed that the statistical properties of the stimuli may influence the nonsymbolic number comparison, such effect was demonstrated here. Importantly, since the size effect modification was mediated by the threshold and/or nondecision time, this size effect change does not invalidate either the ANS account of nonsymbolic comparison, or the related tests of the DSS model. From the viewpoint of the ANS account, nonsymbolic comparison can still be considered a psychophysical process, even if stimulus frequency generates an additional effect to the comparison performance—although this frequency effect should be considered while choosing the measurement methods, as discussed above. From the viewpoint of the hybrid ANS–DSS framework, several studies testing this framework relied on the fact that nonsymbolic comparison is not influenced by the statistical properties of the stimuli. The present results demonstrated that, in line with this assumption, the distance effect is dominantly not lead by the statistical properties of the stimuli. In addition, while the frequency of the stimuli influence the nonsymbolic comparison size effect, the effect is not dominant and it is clearly differentiable from the frequency-based size effect modification in symbolic comparison, since the nonsymbolic and symbolic size effect changes rely on different diffusion parameters. Overall, while the present results enrich the picture about the size effect, they do not invalidate either the ANS account of nonsymbolic comparison, or the hybrid ANS–DSS framework.

The present work investigated the role of the statistical properties of the stimuli in numerical comparison among university students. Since the task relies on a rather elementary psychophysical process, we have no reasons to believe that the current results could not be generalized to other human populations in similar tasks.

To conclude, the present results highlight that (a) symbolic and nonsymbolic number processing is supported by different representations, (b) the seemingly uniform size effect is in fact a combination of various effects, where different stimulus formats involve different combinations of the sources, and (c) stimulus frequency can modify the performance in comparison task which can be relevant in psychophysical measurements.

## Supplementary Information

Below is the link to the electronic supplementary material.

Supplementary file1 (PDF 127 KB)

## Data Availability

All raw data are available at https://osf.io/5rzte/.

## Footnotes

1 While the numerical cognition literature usually refers to the psychophysical process behind the comparison task as Weber’s law, it is more appropriate to use the term Weber principle (Algom, 2021).

2 Note that the connectionist model of Tom Verguts and colleagues relies on the frequency of symbolic numbers to account for the ratio effect in a symbolic comparison task (Verguts & Fias, 2004; Verguts et al., 2005). However, those works were not explicit about whether the model can be considered as an extension or refinement of the ANS model or whether the model is an alternative model which points to phenomena that are incompatible with the ANS model. While that connectionist model has been interpreted as an ANS model (Dehaene, 2007), others argue that since the model cannot explain the ratio effect without adding frequency (i.e., it does not obey Weber’s law), it cannot be considered as a psychophysics-based model (Krajcsi et al., 2016).

3 The logarithm of the distances in these models was chosen so that the present results could be more directly comparable with the similar symbolic number comparison task in previous works (Kojouharova & Krajcsi, 2018; Krajcsi & Kojouharova, 2017), where it was found that distance effect can be described better with the logarithm of the distance than with the distance itself. Still, the present data were analyzed with the linear version of the distance effect as well, and the results showed the very same pattern as with the logarithmic version. The results of the linear version analyses are not reported here in detail.

4 Note that this reasoning is based on the classic diffusion model where it is assumed that the threshold is constant within the trial. However, if the frequency-based manipulation influences the threshold, then the threshold should be modified during the trials (see additional details in the Supplementary material), which is not allowed in the classic model. Still, the present argument can be extended to models with collapsing thresholds as well.

5 It is yet an open question what exact mechanisms lead to the differences listed above. As a possibility, one may imagine that a symbolic and a nonsymbolic value are different in the sense that a symbolic number is denoted by the same symbol, whereas a nonsymbolic value can be displayed in different ways. This difference may lead to constraints on what component(s) of the processes may react to the frequency of the stimuli.

## Code availability

Additional script used in this study are available upon request.

## References

[CR1] Alexandrowicz, R. W. (2020). The diffusion model visualizer: An interactive tool to understand the diffusion model parameters. *Psychological Research,**84*(4), 1157–1165. 10.1007/s00426-018-1112-6 30361811 10.1007/s00426-018-1112-6 PMC7239816

[CR2] Algom, D. (2021). The Weber-Fechner law: A misnomer that persists but that should go away. *Psychological Review,**128*(4), 757–765. 10.1037/rev0000278 34242050 10.1037/rev0000278

[CR3] Anobile, G., Cicchini, G. M., & Burr, D. C. (2014). Separate mechanisms for perception of numerosity and density. *Psychological Science,**25*(1), 265–270. 10.1177/0956797613501520 24270462 10.1177/0956797613501520

[CR4] Brockbank, E., Barner, D., & Vul, E. (2022). Ongoing dynamic calibration produces unstable number estimates. *Journal of Experimental Psychology: General,**151*(9), 2092–2114. 10.1037/xge0001178 35201839 10.1037/xge0001178

[CR5] Brus, J., Heng, J. A., & Polanía, R. (2019). Weber’s law: A mechanistic foundation after two centuries. *Trends in Cognitive Sciences,**23*(11), 906–908. 10.1016/j.tics.2019.09.001 31629634 10.1016/j.tics.2019.09.001

[CR6] Burr, D., & Ross, J. (2008). A visual sense of number. *Current Biology,**18*(6), 425–428. 10.1016/j.cub.2008.02.052 18342507 10.1016/j.cub.2008.02.052

[CR7] Chesney, D. (2018). Numerical distance effect size is a poor metric of approximate number system acuity. *Attention, Perception, & Psychophysics,**80*(5), 1057–1063. 10.3758/s13414-018-1515-x 10.3758/s13414-018-1515-x 29651753

[CR8] Cicchini, G. M., Anobile, G., & Burr, D. C. (2014). Compressive mapping of number to space reflects dynamic encoding mechanisms, not static logarithmic transform. *Proceedings of the National Academy of Sciences,**111*(21), 7867–7872. 10.1073/pnas.1402785111 10.1073/pnas.1402785111 PMC4040572 24821771

[CR9] Cohen Kadosh, R., & Walsh, V. (2009). Numerical representation in the parietal lobes: Abstract or not abstract? *Behavioral and Brain Sciences,**32*(3/4), 313–328. 10.1017/S0140525X09990938 19712504 10.1017/S0140525X09990938

[CR10] Dakin, S. C., Tibber, M. S., Greenwood, J. A., Kingdom, F. A. A., & Morgan, M. J. (2011). A common visual metric for approximate number and density. *Proceedings of the National Academy of Sciences,**108*(49), 19552–19557. 10.1073/pnas.1113195108 10.1073/pnas.1113195108 PMC3241748 22106276

[CR11] Dehaene, S. (1992). Varieties of numerical abilities. *Cognition,**44*, 1–42. 10.1016/0010-0277(92)90049-N 1511583 10.1016/0010-0277(92)90049-n

[CR12] Dehaene, S. (2007). Symbols and quantities in parietal cortex: Elements of a mathematical theory of number representation and manipulation. In P. Haggard, Y. Rossetti, & M. Kawato (Eds.), *Sensorimotor foundations of higher cognition* (22nd ed., pp. 527–574). Harvard University Press.

[CR13] Dehaene, S., & Changeux, J.-P. (1993). Development of elementary numerical abilities: A neural model. *Journal of Cognitive Neuroscience,**5*(4), 390–407. 23964915 10.1162/jocn.1993.5.4.390

[CR14] Dehaene, S., & Mehler, J. (1992). Cross-linguistic regularities in the frequency of number words. *Cognition,**43*, 1–29. 10.1016/0010-0277(92)90030-L 1591901 10.1016/0010-0277(92)90030-l

[CR15] Deneve, S. (2012). Making decisions with unknown sensory reliability. *Frontiers in Neuroscience*, *6*. 10.3389/fnins.2012.00075 10.3389/fnins.2012.00075 PMC3367295 22679418

[CR16] DeWind, N. K., Adams, G. K., Platt, M. L., & Brannon, E. M. (2015). Modeling the approximate number system to quantify the contribution of visual stimulus features. *Cognition,**142*, 247–265. 10.1016/j.cognition.2015.05.016 26056747 10.1016/j.cognition.2015.05.016 PMC4831213

[CR17] Dietrich, J. F., Huber, S., & Nuerk, H.-C. (2015). Methodological aspects to be considered when measuring the approximate number system (ANS)—A research review. *Frontiers in Psychology*, *6*. 10.3389/fpsyg.2015.00295 10.3389/fpsyg.2015.00295 PMC4362052 25852612

[CR18] Evans, N. J., & Hawkins, G. E. (2019). When humans behave like monkeys: Feedback delays and extensive practice increase the efficiency of speeded decisions. *Cognition,**184*, 11–18. 10.1016/j.cognition.2018.11.014 30553935 10.1016/j.cognition.2018.11.014

[CR19] Faul, F., Erdfelder, E., Lang, A.-G., & Buchner, A. (2007). G*Power 3: A flexible statistical power analysis program for the social, behavioral, and biomedical sciences. *Behavioral Research Methods, Instruments and Computers,**39*, 175–191. 10.3758/bf03193146 17695343

[CR20] Gebuis, T., & Reynvoet, B. (2012). The interplay between nonsymbolic number and its continuous visual properties. *Journal of Experimental Psychology: General,**141*(4), 642–648. 10.1037/a0026218 22082115 10.1037/a0026218

[CR21] Giner-Sorolla, R., Montoya, A., Aberson, C., Carpenter, T., Neil Lewis, J., Bostyn, D. H., ..., & Soderberg, C. K. (2023). Power to detect what? Considerations for planning and evaluating sample size. *PsyArXiv Preprints*. 10.31234/osf.io/rv3kw 10.1177/10888683241228328 PMC11193916 38345247

[CR22] Halberda, J., Mazzocco, M. M. M., & Feigenson, L. (2008). Individual differences in non-verbal number acuity correlate with maths achievement. *Nature,**455*(7213), 665–668. 10.1038/nature07246 18776888 10.1038/nature07246

[CR23] Halberda, J., Ly, R., Wilmer, J. B., Naiman, D. Q., & Germine, L. (2012). Number sense across the lifespan as revealed by a massive Internet-based sample. *Proceedings of the National Academy of Sciences,**109*(28), 11116–11120. 10.1073/pnas.1200196109 10.1073/pnas.1200196109 PMC3396479 22733748

[CR24] Hepdarcan, I., Bulut, M., Palaz, E., Can, S., & Dural, S. (2021). The distance effect on discrimination ability and response bias during magnitude comparison in a go/no-go task. *Attention, Perception, & Psychophysics,**83*, 2052–2060. 10.3758/s13414-021-02274-5 10.3758/s13414-021-02274-5 33759115

[CR25] Hohol, M., Willmes, K., Nęcka, E., Brożek, B., Nuerk, H.-C., & Cipora, K. (2020). Professional mathematicians do not differ from others in the symbolic numerical distance and size effects. *Scientific Reports,**10*(1), 1. 10.1038/s41598-020-68202-z 32661271 10.1038/s41598-020-68202-z PMC7359336

[CR26] Kaufman, E. L., Lord, M. W., Reese, T. W., & Volkmann, J. (1949). The discrimination of visual number. *American Journal of Psychology,**62*(4), 498–525. 15392567

[CR27] Kingdom, F. A. A., & Prins, N. (2010). *Psychophysics: A practical introduction*. Academic.

[CR28] Kojouharova, P., & Krajcsi, A. (2018). The Indo-Arabic distance effect originates in the response statistics of the task. *Psychological Research*. 10.1007/s00426-018-1052-1. Advance online publication. 10.1007/s00426-018-1052-1 30030613

[CR29] Kojouharova, P., & Krajcsi, A. (2019). Two components of the Indo-Arabic numerical size effect. *Acta Psychologica,**192*, 163–171. 10.1016/j.actpsy.2018.11.009 30529927 10.1016/j.actpsy.2018.11.009

[CR30] Krajcsi, A. (2021). *CogStat–—An automatic analysis statistical software* (Version 2.1.0) [Computer software]. https://www.cogstat.org

[CR31] Krajcsi, A., & Kojouharova, P. (2017). Symbolic numerical distance effect does not reflect the difference between numbers. *Frontiers in Psychology*, *8*. 10.3389/fpsyg.2017.02013 10.3389/fpsyg.2017.02013 PMC5715324 29250002

[CR32] Krajcsi, A. (2017). Numerical distance and size effects dissociate in Indo-Arabic number comparison. *Psychonomic Bulletin & Review,**24*(8), 927–934. 10.3758/s13423-016-1175-6 27753045 10.3758/s13423-016-1175-6

[CR33] Krajcsi, A. (2020). Ratio effect slope can sometimes be an appropriate metric of the approximate number system sensitivity. *Attention, Perception, & Psychophysics,**82*(4), 2165–2176. 10.3758/s13414-019-01939-6 10.3758/s13414-019-01939-6 PMC7297849 31933171

[CR34] Krajcsi, A., & Kojouharova, P. (2023). Stimulus frequency alone can account for the size effect in number comparison. *Acta Psychologica,**232*, 103817. 10.1016/j.actpsy.2022.103817 36571893 10.1016/j.actpsy.2022.103817

[CR35] Krajcsi, A., Szabó, E., & Mórocz, I. Á. (2013). Subitizing is sensitive to the arrangement of objects. *Experimental Psychology,**60*(4), 227–234. 10.1027/1618-3169/a000191 23422657 10.1027/1618-3169/a000191

[CR36] Krajcsi, A., Lengyel, G., & Kojouharova, P. (2016). The source of the symbolic numerical distance and size effects. *Frontiers in Psychology*, *7*. 10.3389/fpsyg.2016.01795 10.3389/fpsyg.2016.01795 PMC5116562 27917139

[CR37] Krajcsi, A., Lengyel, G., & Kojouharova, P. (2018). Symbolic number comparison is not processed by the analogue number system: Different symbolic and nonsymbolic numerical distance and size effects. *Frontiers in Psychology*, *9*. 10.3389/fpsyg.2018.00124 10.3389/fpsyg.2018.00124 PMC5817629 29491845

[CR38] Krajcsi, A., Kojouharova, P., & Lengyel, G. (2022). Processing symbolic numbers: The example of distance and size effects. In J. Gervain, G. Csibra, & K. Kovács (Eds.), *A life in cognition: Studies in cognitive science in honor of Csaba Pléh* (pp. 379–394). Springer. 10.1007/978-3-030-66175-5_27

[CR39] Krajcsi, A., Chesney, D., Cipora, K., Coolen, I., Gilmore, C., Inglis, M., ..., & Reynvoet, B. (2024a). Measuring the acuity of the approximate number system in young children. *Developmental Review*, *72*, 101131. 10.1016/j.dr.2024.101131

[CR40] Krajcsi, A., Lengyel, G., & Kojouharova, P. (2024b). *A new framework for elementary number processing: The hybrid ANS–DSS account.* Manuscript submitted for publication.

[CR41] Leibovich, T., Katzin, N., Harel, M., & Henik, A. (2017). From “sense of number” to “sense of magnitude”: The role of continuous magnitudes in numerical cognition. *Behavioral and Brain Sciences,**40*, e164. 10.1017/S0140525X16000960 27530053 10.1017/S0140525X16000960

[CR42] Mandler, G., & Shebo, B. J. (1982). Subitizing: An analysis of its component processes. *Journal of Experimental Psychology: General,**111*(1), 1–22. 10.1037/0096-3445.111.1.1 6460833 10.1037//0096-3445.111.1.1

[CR43] Moyer, R. S., & Landauer, T. K. (1967). Time required for judgements of numerical inequality. *Nature,**215*(5109), 1519–1520. 10.1038/2151519a0 6052760 10.1038/2151519a0

[CR44] Nieder, A. (2005). Counting on neurons: The neurobiology of numerical competence. *Nature Reviews Neuroscience,**6*, 177–190. 10.1038/nrn1626 15711599 10.1038/nrn1626

[CR45] Odic, D., & Starr, A. (2018). An introduction to the approximate number system. *Child Development Perspectives,**12*(4), 223–229. 10.1111/cdep.12288 30534193 10.1111/cdep.12288 PMC6286047

[CR46] Odic, D., Hock, H., & Halberda, J. (2014). Hysteresis affects approximate number discrimination in young children. *Journal of Experimental Psychology General,**143*(1), 255–265. 10.1037/a0030825 23163765 10.1037/a0030825 PMC4390026

[CR47] Palmer, J., Huk, A. C., & Shadlen, M. N. (2005). The effect of stimulus strength on the speed and accuracy of a perceptual decision. *Journal of Vision,**5*(5), 1–1. 10.1167/5.5.1 16097871 10.1167/5.5.1

[CR48] Pardo-Vazquez, J. L., Castiñeiras-de Saa, J. R., Valente, M., Damião, I., Costa, T., Vicente, M. I., ..., & Renart, A. (2019). The mechanistic foundation of Weber’s law. *Nature Neuroscience*, *22*(9), 9. 10.1038/s41593-019-0439-7 10.1038/s41593-019-0439-7 31406366

[CR49] Park, J., & Brannon, E. M. (2013). Training the approximate number system improves math proficiency. *Psychological Science,**24*(10), 2013–2019. 10.1177/0956797613482944 23921769 10.1177/0956797613482944 PMC3797151

[CR50] Peirce, J. W. (2007). PsychoPy–Psychophysics software in Python. *Journal of Neuroscience Methods,**162*(1/2), 8–13. 10.1016/j.jneumeth.2006.11.017 17254636 10.1016/j.jneumeth.2006.11.017 PMC2018741

[CR51] Petzschner, F. H., Glasauer, S., & Stephan, K. E. (2015). A Bayesian perspective on magnitude estimation. *Trends in Cognitive Sciences,**19*(5), 285–293. 10.1016/j.tics.2015.03.002 25843543 10.1016/j.tics.2015.03.002

[CR52] Piantadosi, S. T. (2014). Zipf’s word frequency law in natural language: A critical review and future directions. *Psychonomic Bulletin & Review,**21*(5), 1112–1130. 10.3758/s13423-014-0585-6 24664880 10.3758/s13423-014-0585-6 PMC4176592

[CR53] Piantadosi, S. T. (2016). A rational analysis of the approximate number system. *Psychonomic Bulletin & Review,**23*(3), 877–886. 10.3758/s13423-015-0963-8 26755188 10.3758/s13423-015-0963-8 PMC4889539

[CR54] Piazza, M., Facoetti, A., Trussardi, A. N., Berteletti, I., Conte, S., Lucangeli, D., ..., & Zorzi, M. (2010). Developmental trajectory of number acuity reveals a severe impairment in developmental dyscalculia. *Cognition*, *116*(1), 33–41. 10.1016/j.cognition.2010.03.012 10.1016/j.cognition.2010.03.012 20381023

[CR55] Ratcliff, R., & McKoon, G. (2008). The diffusion decision model: Theory and data for two-choice decision tasks. *Neural Computation,**20*(4), 873–922. 10.1162/neco.2008.12-06-420 18085991 10.1162/neco.2008.12-06-420 PMC2474742

[CR56] Ratcliff, R., Smith, P. L., Brown, S. D., & McKoon, G. (2016). Diffusion decision model: Current issues and history. *Trends in Cognitive Sciences,**20*(4), 260–281. 10.1016/j.tics.2016.01.007 26952739 10.1016/j.tics.2016.01.007 PMC4928591

[CR57] Ren, X., & Libertus, M. E. (2024). (Dis)similarities between non-symbolic and symbolic number representations: Insights from vector space models. *Acta Psychologica,**248*, 104374. 10.1016/j.actpsy.2024.104374 38908226 10.1016/j.actpsy.2024.104374

[CR58] Revkin, S. K., Piazza, M., Izard, V., Cohen, L., & Dehaene, S. (2008). Does subitizing reflect numerical estimation? *Psychological Science,**19*(6), 607–614. 18578852 10.1111/j.1467-9280.2008.02130.x

[CR59] Rinaldi, L., Parente, L., & Marelli, M. (2022). Toward a unified account of nonsymbolic and symbolic representations of number: Insights from a combined psychophysical-computational approach. *Psychonomic Bulletin & Review,**29*(3), 985–994. 10.3758/s13423-021-02043-3 34918278 10.3758/s13423-021-02043-3

[CR60] Schneider, M., Beeres, K., Coban, L., Merz, S., Susan Schmidt, S., ..., & De Smedt, B. (2017). Associations of non-symbolic and symbolic numerical magnitude processing with mathematical competence: A meta-analysis. *Developmental Science*, *20*(3). 10.1111/desc.12372 10.1111/desc.12372 26768176

[CR61] Shinn, M., Lam, N. H., & Murray, J. D. (2020). A flexible framework for simulating and fitting generalized drift-diffusion models. *eLife,**9*, e56938. 10.7554/eLife.56938 32749218 10.7554/eLife.56938 PMC7462609

[CR62] Smith, P. L., & Ratcliff, R. (2004). Psychology and neurobiology of simple decisions. *Trends in Neurosciences,**27*(3), 161–168. 10.1016/j.tins.2004.01.006 15036882 10.1016/j.tins.2004.01.006

[CR63] Stoianov, I., & Zorzi, M. (2012). Emergence of a “visual number sense” in hierarchical generative models. *Nature Neuroscience,**15*(2), 194–196. 10.1038/nn.2996 22231428 10.1038/nn.2996

[CR64] Testolin, A., Zou, W. Y., & McClelland, J. L. (2020). Numerosity discrimination in deep neural networks: Initial competence, developmental refinement and experience statistics. *Developmental Science,**23*(5), e12940. 10.1111/desc.12940 31977137 10.1111/desc.12940

[CR65] The Jamovi Project. (2020). *Jamovi* (Version 1.2) [Computer software]. https://www.jamovi.org

[CR66] Verguts, T., & Fias, W. (2004). Representation of number in animals and humans: A neural model. *Journal of Cognitive Neuroscience,**16*(9), 1493–1504. 10.1162/0898929042568497 15601514 10.1162/0898929042568497

[CR67] Verguts, T., Fias, W., & Stevens, M. (2005). A model of exact small-number representation. *Psychonomic Bulletin & Review,**12*(1), 66–80. 10.3758/BF03196349 15945201 10.3758/bf03196349

[CR68] Voss, A., Nagler, M., & Lerche, V. (2013). Diffusion models in experimental psychology. *Experimental Psychology,**60*(6), 385–402. 10.1027/1618-3169/a000218 23895923 10.1027/1618-3169/a000218

[CR69] Wagenmakers, E.-J., van der Maas, H. L. J., & Grasman, R. P. P. P. (2007). An EZ-diffusion model for response time and accuracy. *Psychonomic Bulletin & Review,**14*(1), 3–22. 10.3758/BF03194023 17546727 10.3758/bf03194023

