# Supplementary material for: Different sources of the numerical comparison size effect
Source: Mem Cognit. 2026 Mar 17;54(3):792–812. doi: 10.3758/s13421-025-01781-2 (PMC13132974; doi:10.3758/s13421-025-01781-2)
Supplement: Supplementary file 1 — Supplementary file1 (PDF 127 KB) [file 13421_2025_1781_MOESM1_ESM.pdf]

Supplementary material for the article “Different sources of the numerical comparison size effect”

Attila Krajcsi, Petia Kojouharova

Quantitative diffusion model parameter recovery

Initially, the EZ recovery method was applied (Wagenmakers et al., 2007) to analyze the present data. It can recover three parameters: Drift rate, threshold, and nondecision time. These three parameters should be sufficient for our aims because (a) in the present task, it is unreasonable that the starting point parameter is biased, and (b) the additional variability parameters of the main parameters in the full drift diffusion model have relatively small effects on the responses (Voss et al., 2013). Additionally, in some circumstances, the EZ method could be preferable when the number of trials is small (Lerche et al., 2017).

An initial parameter recovery analysis was performed with the EZ method: The three diffusion parameters were calculated for each participant and for each number pair, and these parameters were used as in the behavioral data analysis, that is, the size effect regressor was fitted to the data of each participant and the weight of the regressor was compared across the three condition groups. The initial analyses showed that the size effect is significantly different between the three conditions for the threshold (larger thresholds for more frequent stimuli), and the nondecision time parameters (smaller nondecision time for more frequent stimuli), but not for the drift rate parameter.

Unfortunately, two methodological issues question the validity of those results (and this is the main reason why detailed results are not reported here).

First, the EZ parameter recovery method cannot handle data sets where error rate is either 0%, 50% or 100%. In those cases, an edge correction is applied, where the error rate is corrected with a half trial, for example, in a dataset with 20 trials, 0% error rate is corrected to 2.5% (which is 1/20/2). In the present dataset, many cells included 0% error rate. Importantly, because the frequency was manipulated, the number pair data cells included differing numbers of trials, therefore, the size of the edge correction was different for different number pairs. Because the size of the edge correction modifies the recovered parameters, the different size of edge correction between various number pairs introduced different size of biases in the recovered parameters, where this bias correlates with the experimental manipulation and the effect of interest. Overall, one cannot be sure that the recovered diffusion parameters reflect a genuine effect of the cognitive processing or a bias of the analysis and design. Contrasting the behavioral data and the recovered parameters also hints that the recovered parameters may come from the bias caused by the different cell sizes. While, in the reaction time, more frequent stimuli are faster to process, in the threshold parameter, more frequent stimuli show larger thresholds. In the diffusion model, with all other parameters unchanged, larger threshold leads to slower and not faster responses. However, in the EZ method, larger trial numbers lead to smaller edge correction which in turn leads to larger threshold. Overall, if other parameters do not play an essential role here, the initial result of recovered threshold values may be rooted in the methodological bias and not in the responses.

To overcome the limitation of the EZ parameter recovery method, it is possible to use other parameter recovery methods that do not rely on edge correction. Therefore, the pyddm package (Shinn et al., 2020) was used to recover the same three parameters that the EZ method recovers. However, in the data sets with 0% error rates, the recovered parameters are unrealistically high. This cannot be resolved by setting upper limits on those parameters because those limit values will be reached more frequently in number pair cells with fewer trials since these cells have a 0% error rate more probably (the chance to make an erroneous trial is lower because there are fewer trials). Overall, the initial issue could not be resolved with this alternative recovery method that does not rely on edge correction. These results also highlight that using the diffusion models in experimental designs with unequal number of trials per cell could result in biased recovered parameters.

Another potential solution is to collapse cells so that they include more trials, which decreases the possibility of having cells with 0% error rate, but, in our dataset, even collapsing cells to the two possible largest cells (i.e., small numbers vs large numbers) includes too many data sets with 0% error rate. A next possible solution to overcome the analysis bias rooted in the different trial numbers between cells is to remove trials from frequent number pair cells so that all cells have the same number of trials. Unfortunately, in this case, depending on the specific method, at least 80% of the trials are dropped. With this method the results become too noisy and unstable, therefore, no difference in the diffusion parameters could be found.

To sum up, the unequal trial numbers per number pairs made the diffusion parameter recovery method potentially biased, and no alternative appropriate recovery analysis solution was found. Even if initial analyses showed that the size effect was mainly rooted in the change of the threshold and nondecision time, but not in the drift rate, the methodological considerations above made these findings invalid.

The second problem with such findings is a possible contradiction between the results and the type of diffusion process that the recovery methods were designed to find. The preliminary diffusion parameter recovery results suggested that the threshold was changed depending on the frequency of the stimuli. Even if the results of the initial analysis may be strongly biased, it is possible that the frequency of the stimuli may indeed change the threshold (see also the qualitative analysis in the main text). Because the specific number pair and, therefore, the frequencies of those stimuli are learned only after the presentation of the stimuli, frequency-dependent threshold means that the threshold should be changed during the trial. However, the widely applied diffusion model that the EZ method also rely on assumes that the threshold is constant throughout the trial (Wagenmakers et al., 2007). At the end, this leads to a contradiction: The method that assumes a constant threshold reveals a phenomenon where the threshold changes during the trial. However, it is possible that the threshold is mostly changed before the accumulated evidence reaches the threshold, and, in that case, recovery methods assuming constant threshold (such as the currently used EZ) may recover the parameters validly. Alternatively, it is possible that even if the threshold is not constant, depending on the nature of the threshold change the recovery methods can capture the parameter changes appropriately. To sum up, if the threshold is not constant in the present paradigm, the EZ method is not designed to reveal this validly, and the results that suggest collapsing threshold should be handled cautiously.

References

Lerche, V., Voss, A., & Nagler, M. (2017). How many trials are required for parameter estimation in diffusion modeling? A comparison of different optimization criteria. *Behavior Research Methods*, *49*(2), 513–537. https://doi.org/10.3758/s13428-016-0740-2

Shinn, M., Lam, N. H., & Murray, J. D. (2020). A flexible framework for simulating and fitting generalized drift-diffusion models. *eLife*, *9*, e56938. https://doi.org/10.7554/eLife.56938

Voss, A., Nagler, M., & Lerche, V. (2013). Diffusion Models in Experimental Psychology. *Experimental Psychology*, *60*(6), 385–402. https://doi.org/10.1027/1618-3169/a000218

Wagenmakers, E.-J., van der Maas, H. L. J., & Grasman, R. P. P. P. (2007). An EZ-diffusion model for response time and accuracy. *Psychonomic Bulletin & Review*, *14*(1), 3–22. https://doi.org/10.3758/BF03194023
